# Supplementary material for: Supercoiling-dependent DNA binding: quantitative modeling and applications to bulk and single-molecule experiments
Source: Nucleic Acids Res. 2023 Nov 24;52(1):59–72. doi: 10.1093/nar/gkad1055 (PMC10783501; doi:10.1093/nar/gkad1055)
Supplement: gkad1055_Supplemental_Files [file gkad1055_supplemental_files.zip › 20231002_TopologyDependentBinding_SUPP.pdf]

Supplementary Information for

Supercoiling-dependent DNA binding: quantitative modeling and applications to bulk and single-molecule experiments

Pauline J. Kolbeck[1,2,a], Miloš Tišma[3,a], Brian T. Analikwu[3], Willem Vanderlinden[1,2,b], Cees Dekker[3,b], and Jan Lipfert[1,2,b]

[1]*Department of Physics and Center for NanoScience, LMU Munich, Amalienstrasse 54, 80799 Munich, Germany*
[2]*Soft Condensed Matter and Biophysics, Department of Physics and Debye Institute for Nanomaterials Science, Utrecht University, Princetonplein 1, 3584 CC Utrecht, The Netherlands*
[3]*Department of Bionanoscience, Kavli Institute of Nanoscience, Delft University of Technology, Delft, The Netherlands*

[a]Equal contribution
[b]Correspondence: W.Vanderlinden@uu.nl, C.Dekker@tudelft.nl, J.Lipfert@uu.nl

**Equimolar DNA mix (µL)**	**TAE buffer (µL)**	**Gel loading dye (µL)**	**[DNA] (µM · bp)**
20	/	/	0.9231
15	5	1	0.6585
10	10	2	0.4185
5	15	3	0.2000
2	20	4	0.0708
1	25	5	0.0298
0.5	25	5	0.0151

Supplementary Table S1: DNA dilution series for gel electrophoresis assays.

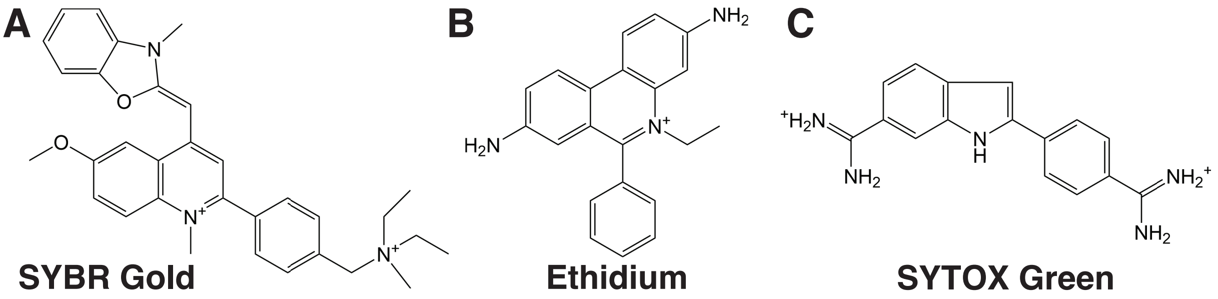

Supplementary Figure S1. Molecular structure of the three intercalators (A) SYBR Gold, (B) Ethidium, (C) SYTOX Green. The SYBR Gold structure was taken from Reference (1), the Ethidium structure from reference (2). Since the structure of SYTOX Orange, the dye used for the single-molecule fluorescence assay in this work, is unknown, we show the structure of the related dye SYTOX Green (taken from reference (3)) here.

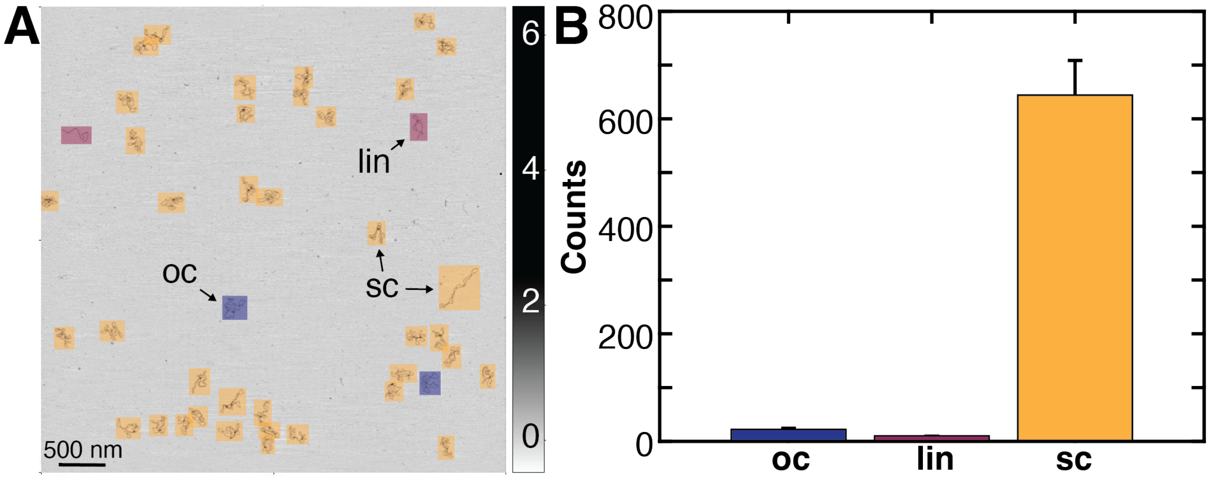

Supplementary Figure S2. DNA topology analysis and quality control via AFM imaging. A) AFM height image of supercoiled pBR322 DNA at a concentration of 1 ng/μL deposited on PLL mica after drying in air. The different topologies are indicated with different colors, open circular DNA (oc) in blue, linear DNA (lin) in red, and supercoiled DNA (sc) in yellow. Z-ranges are indicated in nm by the scale bar on the right. **B)** Topology analysis from AFM experiments of pBR322 DNA that was also used as supercoiled DNA for gel experiments. From a total of 676 molecules, 95% are supercoiled, 3% open circular, and 2% linear. Error bars are from counting statistics.

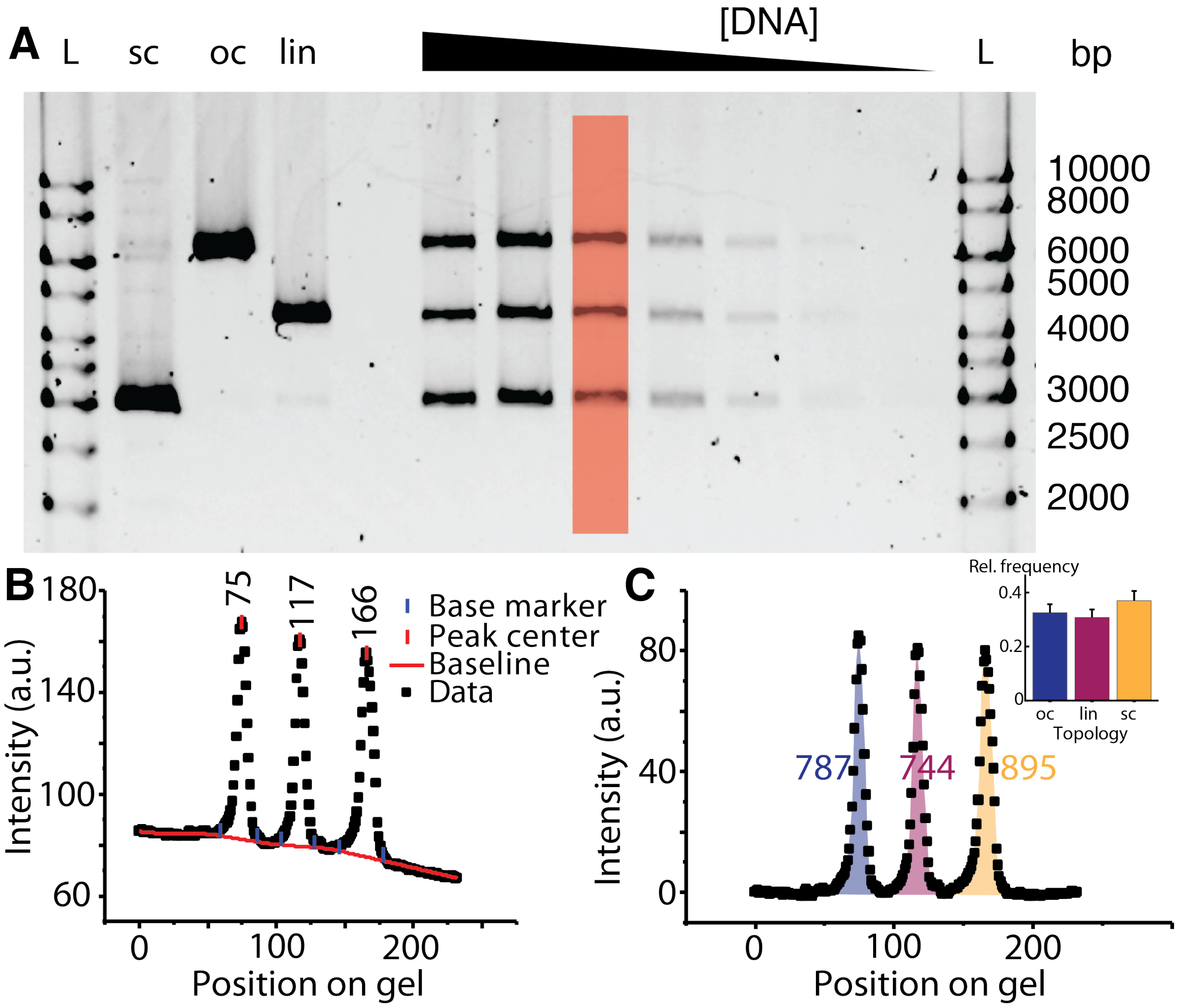

Supplementary Figure S3. Analysis of gel electrophoresis data. A) Agarose gel stained with SYBR Gold at a final concentration of 0.3 µM. Different DNA topologies are separated on the gel. L: DNA size ladders (1 kb gene ruler, Thermo Scientific, 5 µL). Lanes 2-4 are the stock solutions of the supercoiled, linear, and open circular DNA, respectively. Lanes 6-12 are equimolar mixtures of the three topologies, at different total DNA concentrations. For quantitative analysis, individual lanes are selected (as highlighted in red) to create intensity profiles. **B)** Line profile of the area highlighted in red in panel A. Using the software Origin, a baseline is set and the peaks corresponding to the three topologies (“oc”, “lin”, “sc”) are detected automatically. **C)** Same data as in panel B, only baseline corrected. The area under the peaks (which corresponds to the fluorescence intensity of the individual topologies) is calculated and used to determine the intensity ratios of the three topological states. Inset: integrated and normalized intensities of the individual peaks shown in panel C.

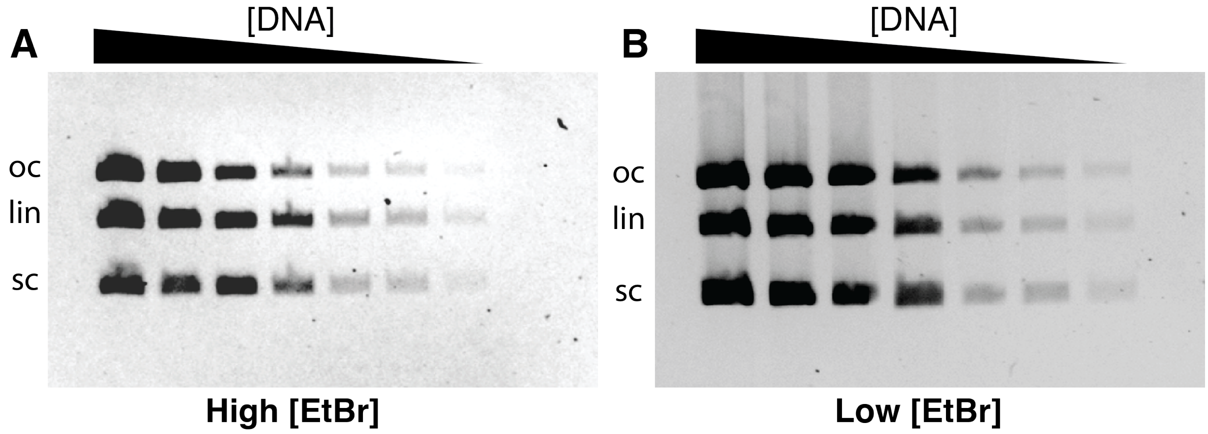

Supplementary Figure S4. Agarose gel stained with EtBr at a final concentration of 5 µM (A) and 0.05 µM (B), respectively. Different DNA topologies are separated on the gel. Lanes 1-7 are equimolar mixtures of the three topologies, at different total DNA concentrations, using the pBR322 plasmid, similar to Figure 2A.

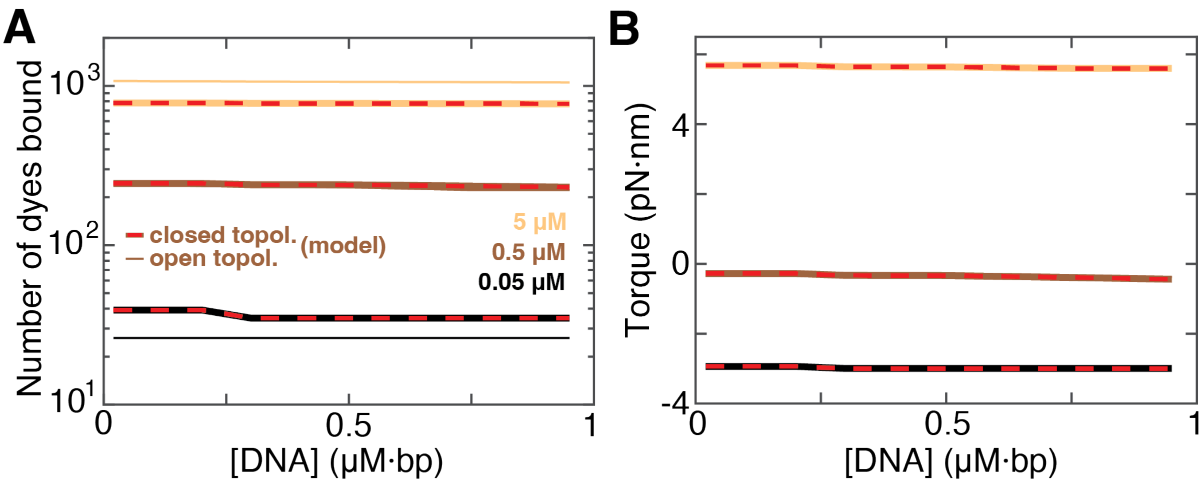

Supplementary Figure S5. Topology dependent binding of EtBr. A) Predicted number of intercalated molecules N_{bound} as function of DNA concentration for pBR322 DNA (4361 bp). Different colors correspond to different EtBr concentrations: from dark to light 0.05, 0.5, and 5 µM. Thin lines are for topologically open DNA (linear and open circular); thick lines with red highlights are for topologically closed DNA (supercoiled, here with initial supercoiling density $\sigma \approx -5\%$, corresponding to $\Delta Lk_0 \approx -20$ turns). The number of molecules bound is approximately independent of DNA concentration under the conditions investigated, but clearly dependents on EtBr concentration and DNA topology. **B)** Predicted torque in the plasmid from our model, same color code as in panel A. The torque in the topologically open conformations is zero and not shown for clarity.

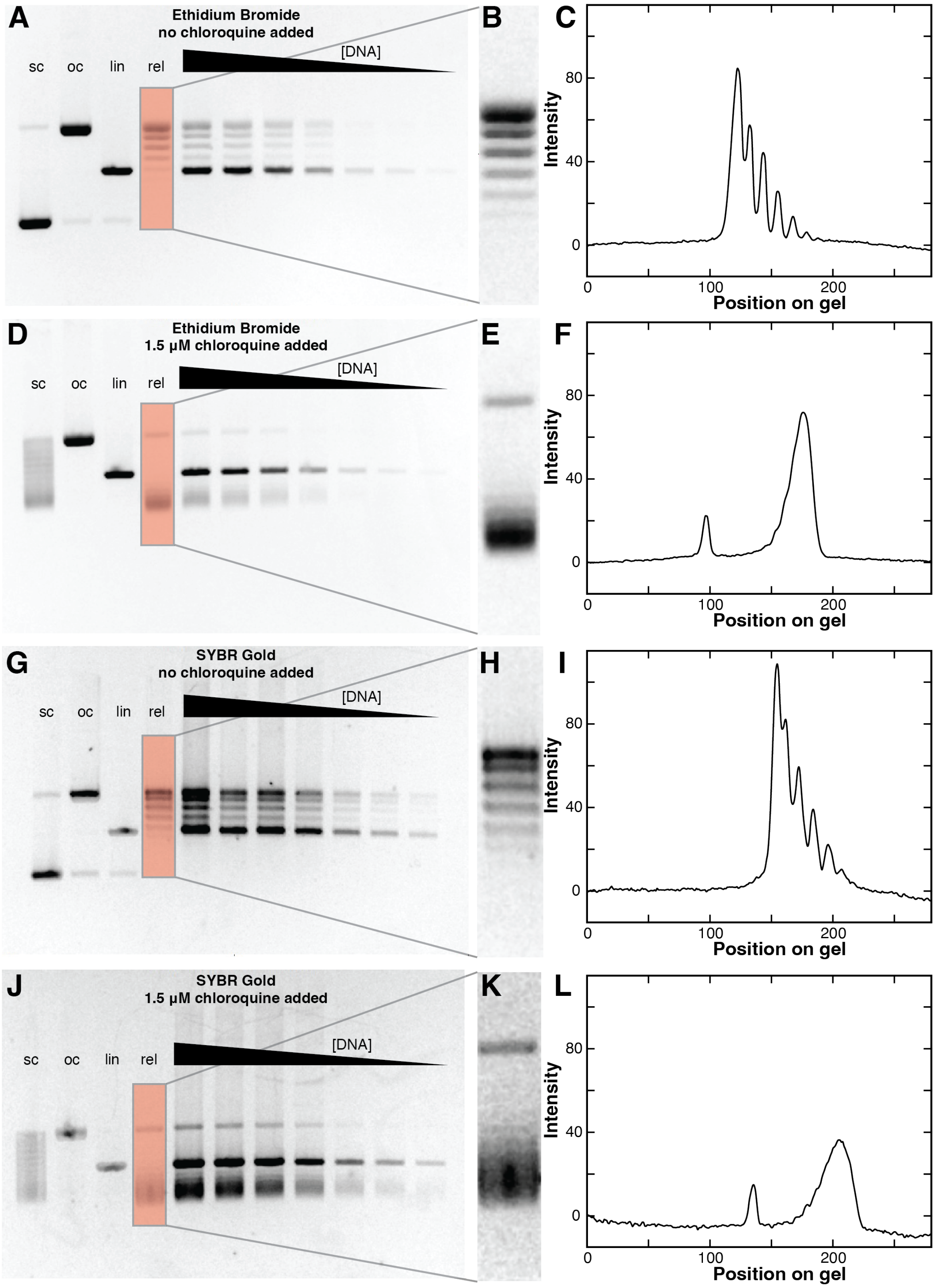

Supplementary Figure S6. Characterization of initially torsionally relaxed plasmids. pBR322 plasmids where relaxed with Wheat Germ Topoisomerase I (Inspiralis) at 37 ºC in a assay buffer containing 50 mM Tris HCl, 1 mM DTT, 20% (v/v) Glycerol, 50 mM NaCl. Subsequently, gel electrophoresis was performed at 22 ºC in a running buffer containing 40 mM Tris, 20 mM NaAc, 1 mM EDTA. **A, D, G, J)** Gels were stained either using EtBr (A,D)

or SYBR Gold (G,J). All gels contained the untreated supercoiled, open circular, and linear DNA for comparisons (lanes 1-3). Lane 4 contains the topoisomerase relaxed plasmid ("rel"). Lanes 5-11 contain equimolar mixtures of relaxed and linear plasmids and are further analyzed in Figure 2 and Supplementary Figure S7. Gels in panels A and G used the regular running buffer. Gels in panel D and J used running buffer supplement with 1.5 μM chloroquine, an intercalator, which will unwind DNA and therefore introduce positive *Wr*. **B, E, H, K)** Zoom into the gel images from panels A, D, G, and J, focusing on the lanes that have the relaxed plasmids only (lane 4). **C, F, I, L)** Lane intensity profile for the lanes that contain the relaxed plasmid only (lane 4). Lane profiles were obtained using the software SPIP (v.6.4, Image Metrology, Hørsholm, Denmark). Electrophoretic analysis of the relaxed plasmid in the absence of chloroquine (A and G) clearly shows individual bands that migrate close to and slightly faster than the oc species, i.e. are close to zero *Wr*. This is expected, since relaxation with topoisomerase I has been show to yield a Gaussian distribution of topoisomers centered on $\Delta Lk = 0$ (4,5). We note that the $\Delta Lk = 0$ topoisomer will have a *Wr* slightly shifted from zero in our gels, since the relaxation reaction and gels used slightly different temperatures and salt conditions and since the DNA twist is temperature (4,6) and salt dependent (7,8). However, we estimate this shift to be only about $\Delta Wr \sim -1$ turn. In the presence of chloroquine (D and J), the topoisomer distribution is shifted to positive *Wr*. Under the condition of our gels, the individual topoisomer bands are not well enough resolved to analyze them using the Depew and Wang approach (5). Nonetheless, it is clear that the relaxed plasmid is close to $\Delta Lk = 0$, with a topoisomer distribution with a standard deviation of at most two ΔLk units, consistent with previous reports (9). For simplicity, we model the "relaxed" plasmid condition using an initial value $\Delta Lk = 0$. Using initial linking numbers in the range $\Delta Lk = \pm 2$ does not significantly change the results in Figure 2D and Figure S7B, within experimental error.

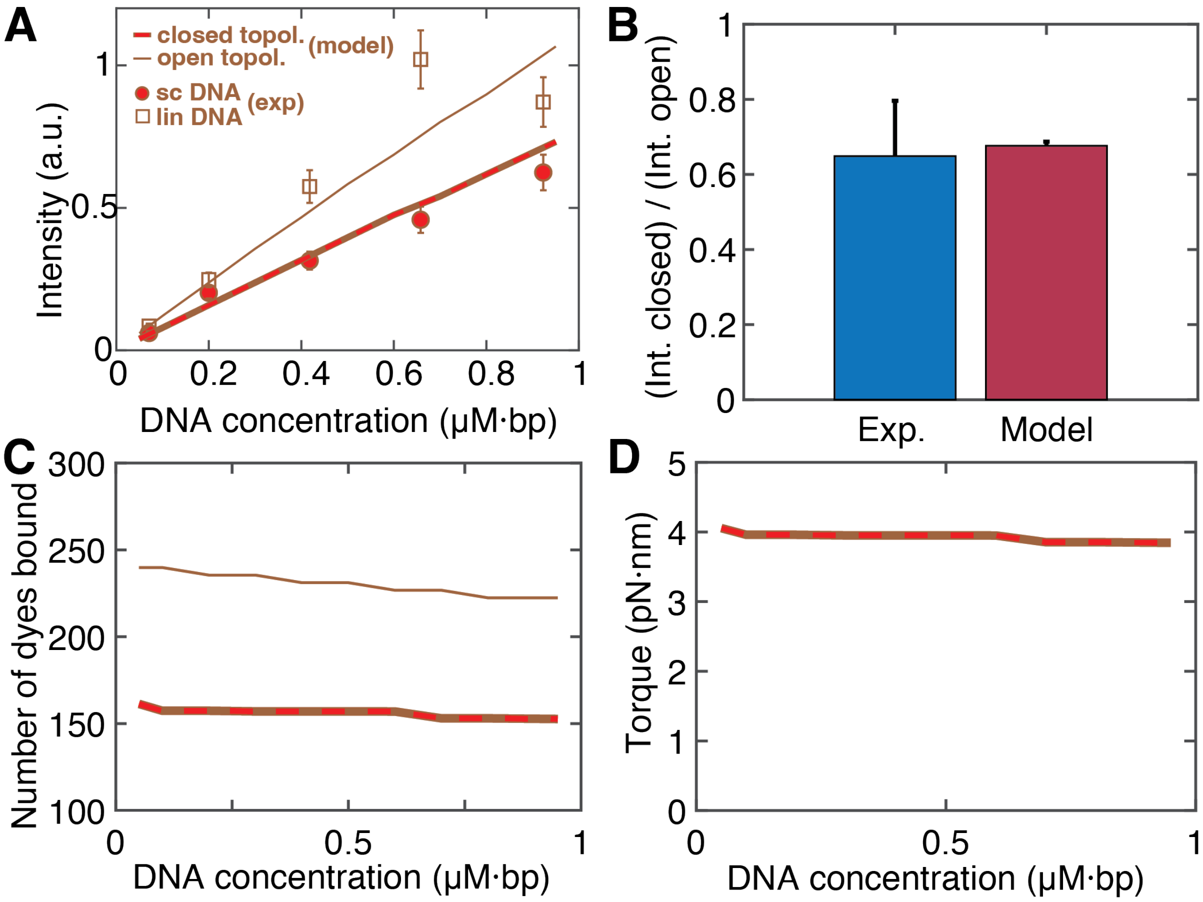

Supplementary Figure S7. Topology dependent binding of EtBr to initially relaxed plasmids. A) Experimentally determined fluorescence intensity for topologically closed DNA (circles with red highlight) and linear DNA (squares) as a function of DNA concentration. The data are taken from the gel shown in Supplementray Figure S6A. Lines are predictions of our binding model (same color code as in Figure 2), with the scale factor α as the only fitting parameter (Equation 7). The EtBr concentration is 0.5 μM. **B)** Relative fluorescence intensity of a topologically closed DNA with $\Delta Lk_0 \approx 0$ relative to the topologically open DNA. The experimental data are the mean and std over different DNA concentration. **C)** Predicted number of intercalated molecules N_{bound} as function of DNA concentration for pBR322 DNA (4361 bp) with $\Delta Lk_0 \approx 0$. The thin line is for the topologically open DNA, the solid thick line with red highlighting for the topologically closed DNA. The number of molecules bound depends only weakly on DNA concentration under the conditions investigated but is clearly reduced for the closed DNA topology compared to linear DNA. **D)** Predicted torque in the plasmid from our model, same color code as in panel A,C.

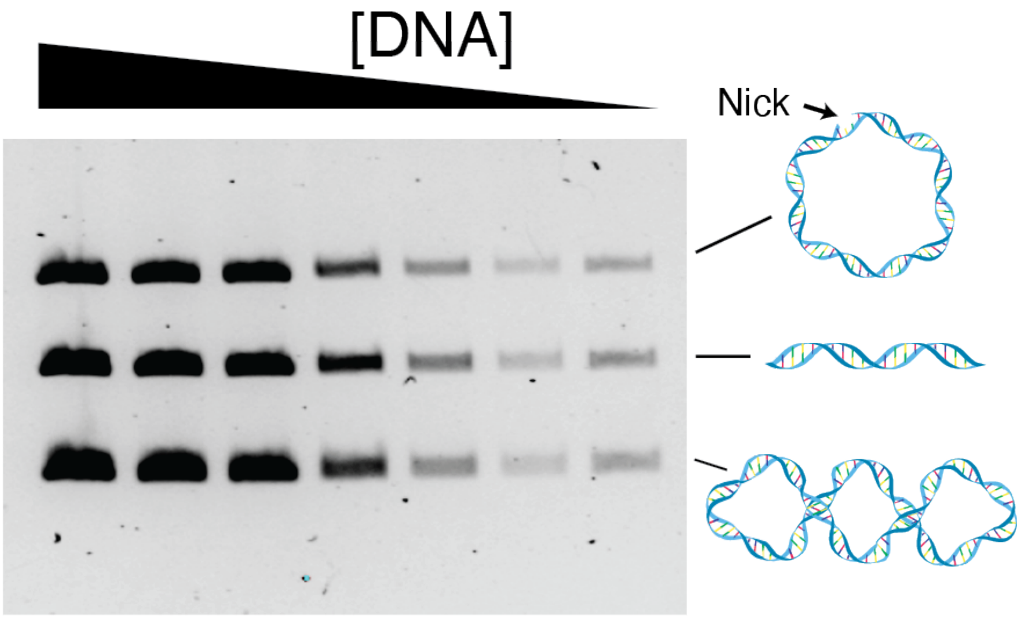

Supplementary Figure S8. Gel electrophoresis with SYBR Gold staining. Agarose gel with equimolar mixtures of open-circular, linear, and supercoiled pBR322 plasmid DNA, stained with SYBR Gold at a final concentration of 0.6 µM.

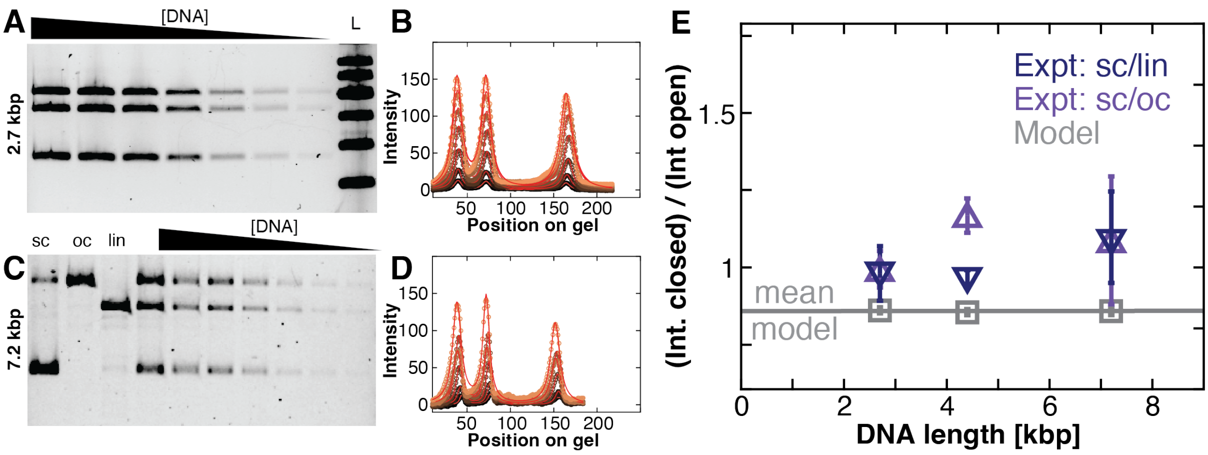

Supplementary Figure S9. Topology dependent intercalation of SYBR Gold for plasmids of different length. A,C) Agarose gels stained with SYBR Gold at a final concentration of 0.6 μM for a 2.7 kbp DNA plasmid (A) and a 7.2 kbp DNA plasmid (C), see Methods for details. Plasmids are natively supercoiled and we assume a supercoiling density of $\sigma \approx -5\%$. The analysis of topology dependent intercalation of SYBR Gold is similar to what is shown in Figure 2A, except for the different DNA length. Lanes with different total DNA concentrations (lanes 1-7 in A and 4-10 in C) contain equimolar mixtures of supercoiled (bottom bands), linear (middle bands), and open circular (top bands) DNA. The ladder in the rightmost lane of panel A is a 1 kb gene ruler (Thermo Scientific) molecular mass standard. **B, D)** Lane intensity profiles of the lanes containing the different topoisomers in A and C, respectively, used for quantification of the intensity for each topological species and DNA concentration. **E)** Relative fluorescence intensity of the topologically closed DNA relative to the topologically open constructs. Experimental data points are obtained by averaging the different DNA concentration and indicate mean ± standard deviation. Gray symbols are the prediction of or model; the gray horizontal line is the mean for the three data points of the model, indicating that the model predicts the binding ratio to be independent of DNA length.

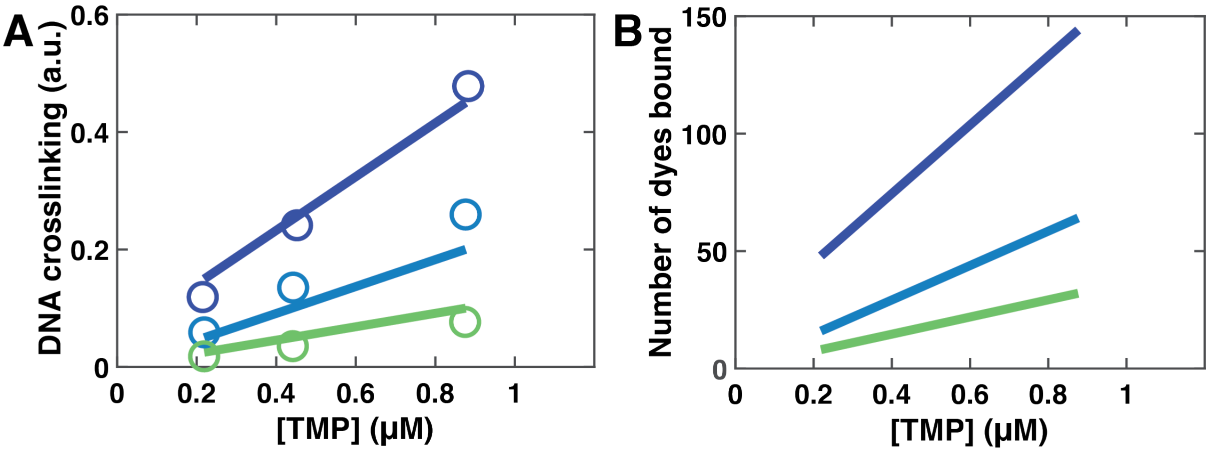

Supplementary Figure S10. Model prediction for TMP binding to plasmid DNA. A) Number of DNA crosslinks as a function of TMP concentration for DNA plasmids with different initial supercoiling density (from blue to green, top to bottom: $\sigma = -0.06, 0, +0.04$). Circles are the experimental data from Ref. (10). Solid lines are the prediction of our model using the parameters in Table 1. B) Number of dyes bound as a function of TMP concentration. Same colour code as in panel A.

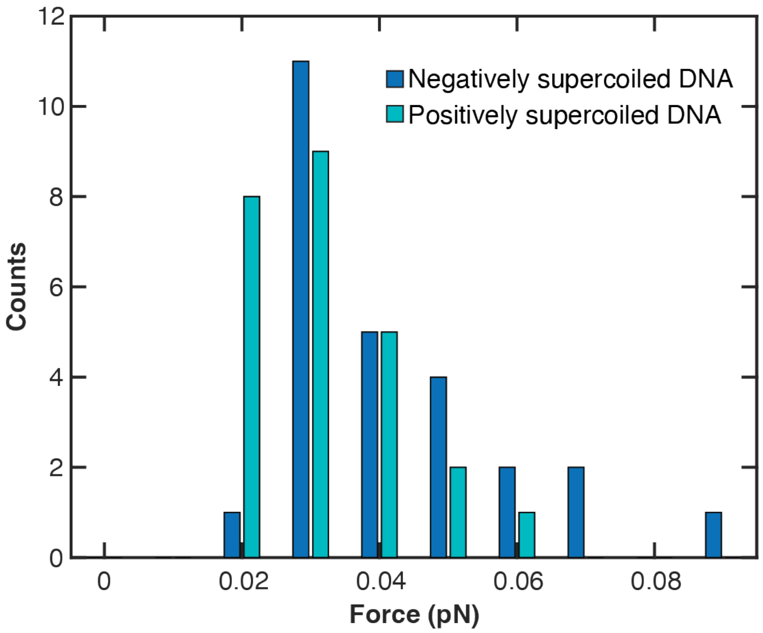

Supplementary Figure S11. Estimate of the stretching forces acting on surface attached DNA molecules in our single-molecule assay. Stretching forces for the surface tethered DNA molecules are estimated from the end-to-end distance (11) using the worm-like chain model (12-14). Despite being stretched due to the surface attachment, the forces are very low, < 0.1 pN. The estimates shown are for nicked molecules, i.e. the effect of supercoiling is not taking into account. However, similar estimates taking into the reduction of the effective contour length due to plectonemic supercoiling still give low forces, < 0.2 pN.

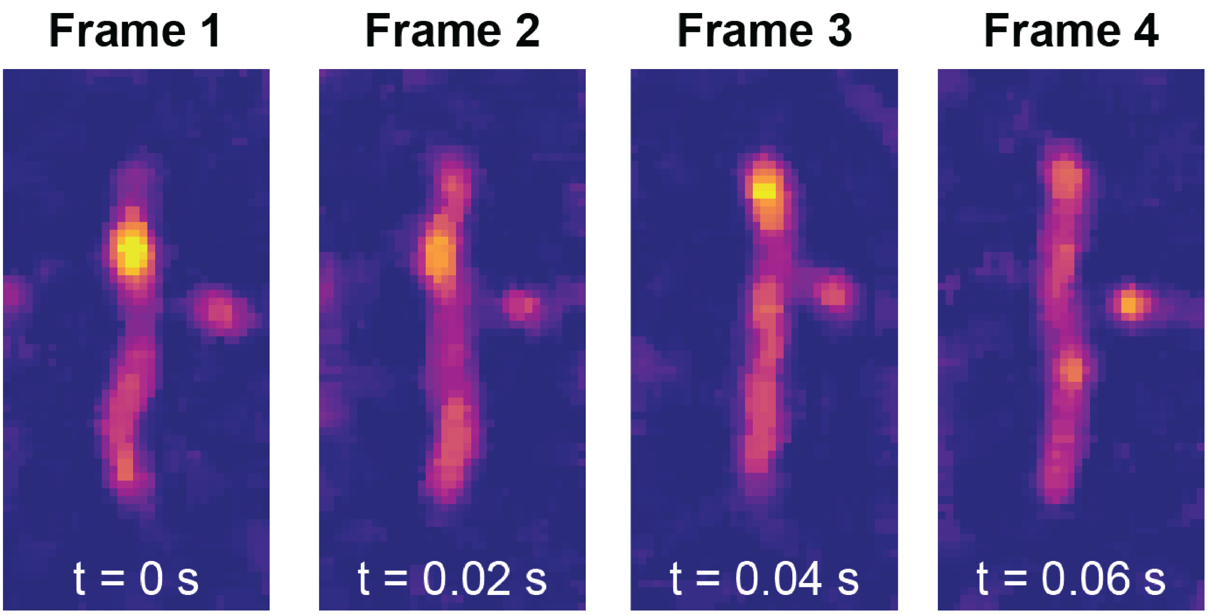

Supplementary Figure S12. Subsequent fluorescence image snapshots at the transition between negatively supercoiled and nicked DNA. We observe that the fluorescent spots disappear over 4 frames (20 ms frame rate), suggesting that writhe relaxation occurs within approximately 80 ms.

Supplementary Movie S1. Movie of initially negatively supercoiled DNA stained by SYTOX Orange. The DNA molecule was attached in the presence of 250 nM SYTOX Orange and the dye concentration was reduced to 50 nM to induce negative supercoils for imaging (see Methods for DNA preparation and imaging parameters). The movie is 2-fold sped up, the time is indicated in the top left corner. Fluctuating bright spots correspond to plectonemes. At around 40 s, the molecule nicks, which leads to an overall decrease of the fluorescence intensity and to the disappearance of the bright spots, in agreement with our model (see main text).

Supplementary Movie S2. Movie of initially positively supercoiled DNA stained by SYTOX Orange. The DNA molecule was attached in the presence of 25 nM SYTOX Orange and the dye concentration was increased to 250 nM to induce positive supercoils for imaging (see Methods for DNA preparation and imaging parameters). The movie is 2-fold sped up, the time is indicated in the top left corner. Fluctuating bright spots correspond to plectonemes. At around 40 s, the molecule nicks, which leads to an overall increase of the fluorescence intensity and to the disappearance of the spots, in agreement with our model (see main text).

Supplementary Movies S3 and S4. Direct visualization of plectonemic DNA under a side flow. DNA molecules were attached in the presence of 25 nM SYTOX Orange and the dye concentration was increased to 250 nM to induce positive supercoils for imaging (see Methods). The movie is 1.5-fold sped up; time is indicated in the top left corner. Fluctuating bright spots, representing moving plectonemes, are visible before and after the strong slide flow is applied. After initial imaging without flow, a strong side flow (11) is applied using a syringe and a second outlet (see Methods; flow is applied between ≈ 6 s and ≈ 25 s), which extrudes the moving bright spots into a single large plectoneme to the side (i.e. towards the top of the field of view).

SUPPLEMENTARY REFERENCES

1. Kolbeck, P.J., Vanderlinden, W., Gemmecker, G., Gebhardt, C., Lehmann, M., Lak, A., Nicolaus, T., Cordes, T. and Lipfert, J. (2021) Molecular structure, DNA binding mode, photophysical properties and recommendations for use of SYBR Gold. *Nucleic Acids Res*, **49**, 5143-5158.
2. Lipfert, J., Klijnhout, S. and Dekker, N.H. (2010) Torsional sensing of small-molecule binding using magnetic tweezers. *Nucleic Acids Res*, **38**, 7122-7132.
3. Wright, D.A. and Welschmeyer, N.A. (2015) Establishing benchmarks in compliance assessment for the ballast water management convention by port state control. *Journal of Marine Engineering & Technology*, **14**, 9-18.
4. Duguet, M. (1993) The helical repeat of DNA at high temperature. *Nucleic Acids Res*, **21**, 463-468.
5. Depew, D. and Wang, J.C. (1975) Conformational fluctuations of DNA helix. *Proceedings of the National Academy of Sciences*, **72**, 4275-4279.
6. Kriegel, F., Matek, C., Drsata, T., Kulenkampff, K., Tschirpke, S., Zacharias, M., Lankas, F. and Lipfert, J. (2018) The temperature dependence of the helical twist of DNA. *Nucleic Acids Res*, **46**, 7998-8009.
7. Cruz-León, S., Vanderlinden, W., Müller, P., Forster, T., Staudt, G., Lin, Y.-Y., Lipfert, J. and Schwierz, N. (2022) Twisting DNA by salt. *Nucleic Acids Res*, **50**, 5726-5738.
8. Anderson, P. and Bauer, W. (1978) Supercoiling in closed circular DNA: dependence upon ion type and concentration. *Biochemistry*, **17**, 594-601.
9. Geggier, S., Kotlyar, A. and Vologodskii, A. (2011) Temperature dependence of DNA persistence length. *Nucleic Acids Res*, **39**, 1419-1426.
10. Bermúdez, I., García-Martínez, J., Pérez-Ortín, J.E. and Roca, J. (2010) A method for genome-wide analysis of DNA helical tension by means of psoralen–DNA photobinding. *Nucleic Acids Res*, **38**, e182-e182.
11. Ganji, M., Shaltiel, I.A., Bisht, S., Kim, E., Kalichava, A., Haering, C.H. and Dekker, C. (2018) Real-time imaging of DNA loop extrusion by condensin. *Science*, **360**, 102-105.
12. Bustamante, C., Marko, J.F., Siggia, E.D. and Smith, S. (1994) Entropic elasticity of lambda-phage DNA. *Science*, **265**, 1599-1600.
13. Bouchiat, C., Wang, M.D., Allemand, J.F., Strick, T., Block, S.M. and Croquette, V. (1999) Estimating the Persistence Length of a Worm-Like Chain Molecule from Force-Extension Measurements. *Biophysical Journal*, **76**, 409-413.
14. Davidson, I.F., Barth, R., Zaczek, M., van der Torre, J., Tang, W., Nagasaka, K., Janissen, R., Kerssemakers, J., Wutz, G., Dekker, C. *et al.* (2023) CTCF is a DNA-tension-dependent barrier to cohesin-mediated loop extrusion. *Nature*, **616**, 822-827.
